# Ki67 Is an Independent Predictor of Recurrence in the Largest Randomized Trial of 3 Radiation Fractionation Schedules in Localized Prostate Cancer

**DOI:** 10.1016/j.ijrobp.2018.01.072

**Published:** 2018-06-01

**Authors:** Anna C. Wilkins, Barry Gusterson, Zsolt Szijgyarto, Joanne Haviland, Clare Griffin, Christine Stuttle, Frances Daley, Catherine M. Corbishley, David P. Dearnaley, Emma Hall, Navita Somaiah, CHHiP Trial Investigators

**Affiliations:** ∗ Institute of Cancer Research-Clinical Trials and Statistics Unit, The Institute of Cancer Research, London, United Kingdom; † Division of Radiotherapy and Imaging, The Institute of Cancer Research, London, United Kingdom; § Division of Breast Cancer Research, The Institute of Cancer Research, London, United Kingdom; ‡ The Royal Marsden, Sutton, London, United Kingdom

## Abstract

**Purpose:**

To assess whether the cellular proliferation marker Ki67 provides prognostic information and predicts response to radiation therapy fractionation in patients with localized prostate tumors participating in a randomized trial of 3 radiation therapy fractionation schedules (74 Gy/37 fractions vs 60 Gy/20 fractions vs 57 Gy/19 fractions).

**Methods and Materials:**

A matched case–control study design was used; patients with biochemical/clinical failure >2 years after radiation therapy (BCR) were matched 1:1 to patients without recurrence using established prognostic factors (Gleason score, prostate-specific antigen, tumor stage) and fractionation schedule. Immunohistochemistry was used to stain diagnostic biopsy specimens for Ki67, which were scored using the unweighted global method. Conditional logistic regression models estimated the prognostic value of mean and maximum Ki67 scores on BCR risk. Biomarker–fractionation interaction terms determined whether Ki67 was predictive of BCR by fractionation.

**Results:**

Using 173 matched pairs, the median for mean and maximum Ki67 scores were 6.6% (interquartile range, 3.9%-9.8%) and 11.0% (interquartile range, 7.0%-15.0%) respectively. Both scores were significant predictors of BCR in models adjusted for established prognostic factors. Conditioning on matching variables and age, the odds of BCR were estimated to increase by 9% per 1% increase in mean Ki67 score (odds ratio 1.09; 95% confidence interval 1.04-1.15, *P* = .001). Interaction terms between Ki67 and fractionation schedules were not statistically significant.

**Conclusions:**

Diagnostic Ki67 did not predict BCR according to fractionation schedule in CHHiP; however, it was a strong independent prognostic factor for BCR.

**Summary**

Radiation therapy is delivered using uniform fractionation for localized prostate tumors despite varying recurrence rates. Biomarkers to guide treatment stratification and predict fraction size sensitivity are needed. This study evaluated Ki67 in localized prostate cancer, for the first time using an internationally validated methodology accounting for intratumoral heterogeneity. Ki67 did not predict recurrence according to fractionation, providing reassurance that hypofractionated schedules can be safely administered in highly proliferative tumors. Ki67 predicted biochemical/clinical recurrence independently of established prognostic factors, including Gleason score.

## Introduction

Prostate cancer (PCa) is the second most common cancer worldwide for males; more than 1.11 million new cases were diagnosed in 2012 (1). In the developed world, increased prostate-specific antigen (PSA) testing means that most patients are diagnosed with localized disease, for which external beam radiation therapy (EBRT), brachytherapy, and prostatectomy are important radical treatment options.

Recurrence rates after EBRT for localized PCa vary considerably, from approximately 10% to 40-50% 2, 3. Recurrences are inadequately predicted using current prognostic algorithms that incorporate Gleason grade, T stage, and presenting PSA level. Identification of prognostic biomarkers to aid treatment stratification would therefore be clinically useful.

In addition, EBRT is delivered using a uniform fractionation schedule for all localized PCa (ie, a “one size fits all approach”). This is despite a wide variation in the biology of localized PCa (4), including proliferation rate (5). A personalized approach to fractionation therefore offers considerable potential to improve therapeutic outcomes. Biomarkers predicting sensitivity to radiation therapy fraction size have recently been identified as a key area for radiobiological research (6).

There is a tight inverse association between the proliferative indices of normal tissues and fractionation sensitivity. Tissues with high proliferation indices, such as gastrointestinal mucosa and epidermis, are insensitive to fraction size. In contrast, late-reacting normal tissues, such as kidney, have low proliferative indices and are very sensitive to fraction size 7, 8. This study tests the hypothesis that the same association between proliferative indices and fractionation sensitivity in normal tissues extends to localized PCa.

The CHHiP trial randomly assigned 3216 men to conventional fractionation (74 Gy in 37 fractions over 7.4 weeks) or 1 of 2 hypofractionated schedules (60 Gy in 20 fractions over 4 weeks or 57 Gy in 19 fractions over 3.8 weeks) (3). Trans-CHHiP is the main translational substudy within CHHiP; tissue blocks from more than 2000 patients have been collected. It provides an excellent opportunity to test the above hypothesis. The expectation is that highly proliferative cancers will show insensitivity to fraction size and be more likely to relapse after the reduced total dose in hypofractionated (>2 Gy) schedules. In contrast, slowly proliferating tumors are expected to be sensitive to fraction size, hence more likely to relapse after conventional fractionation (2 Gy) schedules (7).

## Methods and Material

### Study design

A matched case–control methodology was used to select study participants. The study was approved by the London Multi-centre Research Ethics Committee (04/MRE02/10) and the local ethics committees of all participating centers. Patients experiencing recurrence (cases) were matched 1:1 to patients without recurrence (controls). Matching criteria included fractionation schedule (74 Gy/37 fractions, 60 Gy/20 fractions or 57 Gy/19 fractions) and established prognostic factors including PSA (<10/10-20/>20 ng/mL), Gleason grade (3 + 3/3 + 4/4 + 3/≥4 + 4), and T stage (T1/T2/T3). All tissue samples were centrally reviewed by a specialist uropathologist (C.M.C.), including assignment of Gleason grade according to recent International Society of Urological Pathology and World Health Organization recommendations 9, 10. The centrally assigned Gleason grade was used for matching.

### Immunohistochemistry staining and scoring

Full-face sections from the diagnostic biopsy blocks were used for immunohistochemistry staining. This decision followed a pilot study that demonstrated construction of tissue microarray, using the checkerboard technique (11), resulted in inadequate tumor cellularity (Tables E1 and E2; available online at www.redjournal.org). Immunohistochemistry staining methods are outlined in the Appendix (available online at www.redjournal.org). All slides were scored using bright-field microscopy by 2 independent investigators blinded to recurrence status and fractionation schedule. The CK5/6 basal marker distinguished preinvasive from invasive disease. Prostatic intraepithelial neoplasia and intraductal carcinoma were not scored. A minimum of 100 tumor cells were required to score each case.

**Fig. 1:**
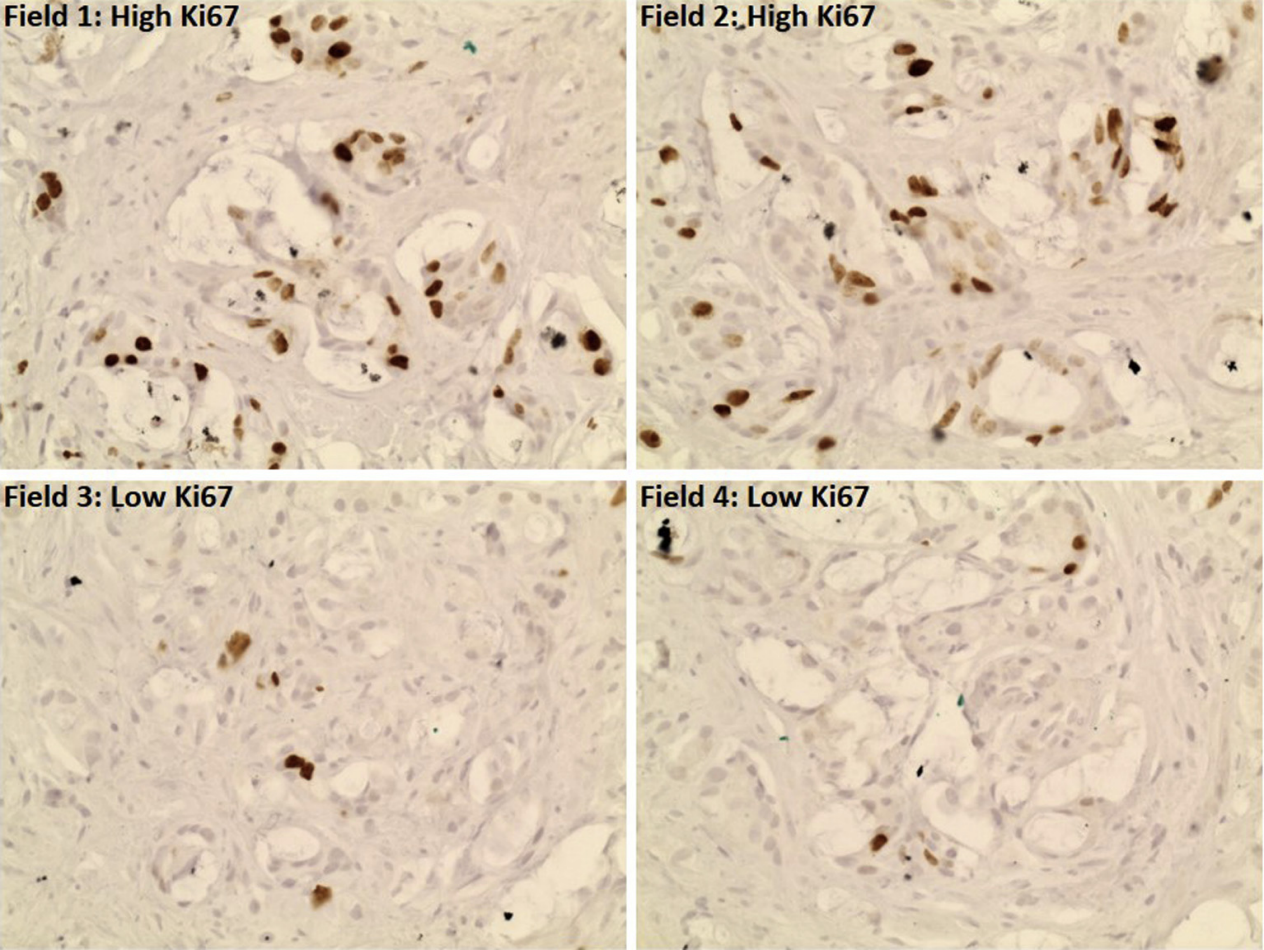
Scoring Ki67 using the global unweighted method. This case contained 50% high proliferation tumor and 50% low proliferation tumor; therefore, high-power fields were selected for 2 highly proliferative areas and 2 low proliferative areas. In this case the numbers of positive staining cells were as follows: field 1, 39/100; field 2, 33/100; field 3, 5/100; and field 4, 5/100. This gives a mean Ki67 score of 20.5% and a maximum Ki67 score of 39%.

The unweighted global assessment of Ki67 developed by the International Ki67 Working Group was used to score all prostate biopsies 12, 13. This includes assessment of intratumoral spatial heterogeneity, which is well recognized in localized PCa (14). The global assessment has met prespecified criteria for scoring reproducibility in an international phase 3 study using core biopsies of breast tumors (13). It involves counting 100 tumor cells in up to 4 high-power fields to derive a mean Ki67 score (Fig. 1). Fields are chosen following an assessment of overall heterogeneity in staining. The final mean Ki67 score consisted of the average of the 2 scoring investigators' mean Ki67 scores for each case. Maximum Ki67 was assessed by 1 investigator and consisted of the highest-scoring individual field (Fig. 1). This was included because the highest proliferative tumor area may be important for radiation therapy response.

All cases with a discrepancy in initial mean Ki67 score >10% were rescored (15). Further rescores were carried out if the discrepancy remained >10%.

### Study endpoints

Mean and maximum Ki67 scores were evaluated. Recurrence was defined as patients with biochemical (16) or clinical failure after radiation therapy (BCR). Patients experiencing BCR within 2 years of radiation therapy commencement were excluded because they are more likely to have developed distant metastases than local recurrence due to radiation therapy failure (2). All data pertaining to recurrence was taken from a CHHiP data snapshot (November 9, 2015) in which median follow-up was 62.4 months (interquartile range, 53.9-77.0 moths). Nonrecurrence was defined in patients with no evidence of BCR alive at the data snapshot.

### Statistical analysis

Agreement in Ki67 scores between the 2 scoring investigators was assessed using Bland-Altman plots to measure the difference between the scores versus the mean of the mean Ki67 scores (17). The concordance correlation coefficient was used to quantify agreement. The difference in the mean Ki67 scores (mean and maximum) between the matched cases and controls by fractionation schedule was compared using paired *t* tests.

Both Ki67 endpoints were analyzed as continuous variables to maximize statistical power (18). Multivariable conditional logistic regression models were fitted to estimate the prognostic value of Ki67 on the risk of BCR, using the entire Trans-CHHiP case–control study cohort. To determine whether Ki67 predicted BCR by fractionation, a biomarker–fractionation interaction term was included. Three comparisons were undertaken to avoid confounding by different recurrence rates across trial arms (74 Gy/37 fractions vs 60 Gy/20 fractions, 74 Gy/37 fractions vs 57 Gy/19 fractions, and 60 Gy/20 fractions vs 57 Gy/19 fractions). Based on an alpha of 0.017, we estimated a power of 75.5%, 74.8%, and 70.0% to detect an interaction between each fractionation schedule and Ki67, respectively (Table E3; available online at www.redjournal.org).

All statistical analysis was conducted using STATA version 13.0 (StataCorp, College Station, TX) and R (version i3863.3.3; R Foundation for Statistical Computing, Vienna).

## Results

### Patient characteristics

A total of 437 cases were assessed by both scoring investigators. Ki67 scores were provided by both investigators in 400 cases; in 37 cases there was insufficient tumor present. The final matched dataset comprised 173 patients with BCR after start of radiation therapy (cases) and 173 patients without recurrence (controls). Matching was achieved to 100% of relevant criteria in all cases analyzed. Fifty-four patients were excluded because they did not have an appropriate match; these were usually controls with no available matching case. Table 1 shows the distribution of the matching variables and age for the controls and cases.

**Table 1:** Distribution of the matching variables and age, according to fractionation schedule

Variable	74 Gy (n = 116, 33.5%)			60 Gy (n = 104, 30.1%)			57 Gy (n = 126, 36.4%)		
	Controls (n = 58)	Cases (n = 58)	%	Controls (n = 52)	Cases (n = 52)	%	Controls (n = 63)	Cases (n = 63)	%
	n	n		n	n		n	n	
PSA (ng/mL)									
<10	17	17	29.3	20	20	38.5	33	33	52.4
10-<20	37	37	63.8	27	27	51.9	26	26	41.3
20	4	4	6.9	5	5	9.6	4	4	6.3
Tumor stage									
T1	11	11	19.0	8	8	15.4	20	20	31.7
T2	42	42	72.4	38	38	73.1	35	35	55.6
T3	5	5	8.6	6	6	11.5	8	8	12.7
Gleason score									
≤6	6	6	10.3	5	5	9.6	8	8	12.7
3+4	31	31	53.4	30	30	57.7	30	30	47.6
4+3	16	16	27.6	10	10	19.2	15	15	23.8
≥8	5	5	8.6	7	7	13.5	10	10	15.9
Age at randomization (y), mean (SD)	69.4 (6.3)	69.8 (6.6)		68.9 (5.4)	68.0 (5.8)		70.1 (6.1)	68.4 (6.2)	
PSA (ng/mL) median (IQR)	12 (8.9-15.8)	11.9 (9.1-16.2)		12.2 (8.6-15.1)	11.6 (8.6-18.1)		9.6 (7.3-13.0)	9.7 (7.2-15.0)	

*Abbreviations:* IQR = interquartile range; PSA = prostate-specific antigen.

### Agreement in Ki67 scores

Of the total of 400 cases scored by both investigators, in 12 cases (3.0%) the difference in mean Ki67 between the 2 scoring investigators was ≥10% (interquartile range, 11.8%-15.1%). These were rescored by both scoring investigators. All rescores were within the required <10% discrepancy.

**Fig. 2:**
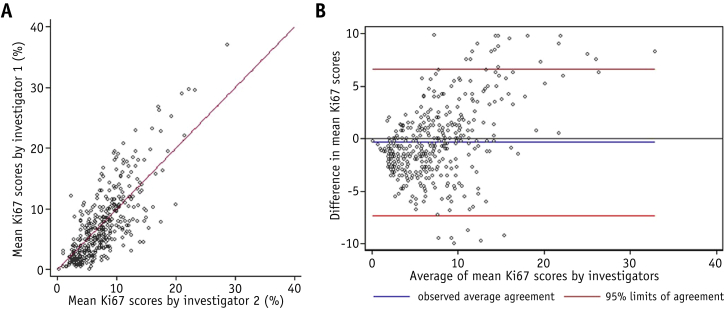
(A) Scatter plot showing concordance in final mean Ki67 between independent scoring investigators. (B) Brand-Altman plot showing difference in final scores between investigator 1 and investigator 2 versus mean scores.

Scatter plots comparing each scoring investigator's final scores, and Bland-Altman plots comparing the difference in final score versus the mean Ki67, are shown in Figure 2 (original scores, Fig. E1; available online at www.redjournal.org). The Bland-Altman plots indicate that the difference in score tended to increase as the mean Ki67 score increased. The overall agreement was considered to be good, with a concordance correlation coefficient of 0.74 (95% confidence interval [CI] 0.70-0.78, *P* < .001) for the final scores. For the original scores before rescore, the concordance correlation coefficient was 0.63 (95% CI 0.58-0.68, *P* < .001).

### Prediction of biochemical/clinical recurrence

**Fig. 3:**
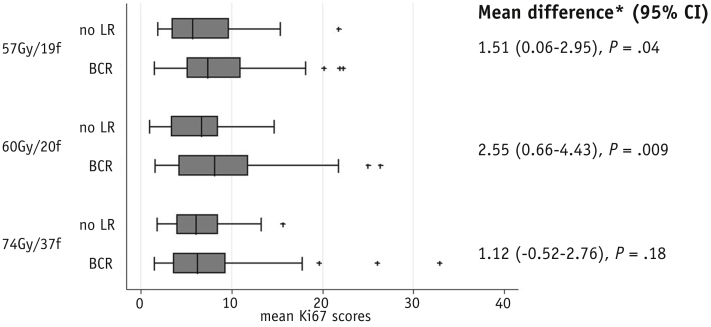
Box and whisker plot showing relationship between mean Ki67 and Gleason grade group.

Multivariable conditional logistic regression models using the entire Trans-CHHiP case–control study sample showed that both mean Ki67 and maximum Ki67 were statistically significant predictors of BCR (Table 2 and Table E4; available online at www.redjournal.org). For each unit increase in mean Ki67 the odds of BCR is estimated to increase by 9% (odds ratio 1.09, 95% CI 1.04-1.15, *P* = .001), having adjusted for matching variables and age. It is clinically relevant that the prediction of recurrence by mean Ki67 is independent of Gleason grade. The lack of correlation between mean Ki67 and Gleason grade is also displayed in the box and whisker plot (Fig. 3). For the maximum Ki67, the odds of BCR were estimated to increase by 5% for each unit increase in the maximum Ki67 score (odds ratio 1.05, 95% CI: 1.01-1.09, *P* = .006) in the multivariable model.

**Table 2:** Odds ratio (OR) for BCR estimated by multivariable conditional logistic regression models (n = 346) using Ki67 as a continuous variable, for mean and maximum Ki67

Ki67 biomarker	OR∗	95% CI	*P*
Mean Ki67 scores	1.09	1.04-1.15	.001
Maximum Ki67 scores	1.05	1.01-1.09	.006

*Abbreviations:* BCR = biochemical or clinical failure after radiation therapy; CI = confidence interval.

∗ Odds ratios are adjusted for the matching variables and age at randomization.

### Prediction of fraction sensitivity

The results of interaction tests between either mean or maximum Ki67 and fractionation schedule were not statistically significant for all comparisons (Table 3 and Table E4; available online at www.redjournal.org). The distribution of mean and maximum Ki67 scores according to fractionation schedule and recurrence status, including a statistical comparison of the difference between cases and controls within fractionation arms, is shown in Figure E2 (available online at www.redjournal.org).

**Table 3:** Odds ratio for BCR estimated from multivariable conditional logistic regression models without and with interaction terms between the mean Ki67 scores and fractionation schedules

Schedules	Variable	OR	95% CI (OR)	*P* (OR)	*P* for interaction∗
74 Gy & 60 Gy	Mean Ki67	1.09	1.02-1.17	.007	.26
74 Gy & 57 Gy	Mean Ki67	1.07	1.01-1.14	.03	.59
60 Gy & 57 Gy	Mean Ki67	1.11	1.04-1.19	.001	.59

Abbreviations as in Table 2.

Odds ratios are adjusted for matching variables and age at randomisation.

∗ *P* value for the interaction between the mean Ki67 scores and fractionation schedules.

## Discussion

This study measures Ki67 staining indices in localized PCa treated with different radiation therapy fractionation schedules. It indicates that the global unweighted method for scoring Ki67 can be used with good agreement between independent scoring investigators without prior experience of this method. To our knowledge this is the first report using the global unweighted method in PCa. However, it is an established method to aid treatment stratification in breast cancer (19), in which Ki67 is used clinically to distinguish between low proliferation luminal A and higher proliferative luminal B breast cancer subtypes (20).

The statistically significant association between mean Ki67 and prediction of BCR has potential clinical application. Our results require external validation in additional patient cohorts, with particular attention to the spectrum of Ki67 expression in different risk groups and a rigorous assessment of scoring concordance in prostate biopsies across different centers. Patients with high mean Ki67 but otherwise lower risk factors could be recommended longer or more intensive androgen deprivation therapy (ADT), with possible addition of docetaxel or abiraterone (21). Patients with low mean Ki67 could be reassured that they are likely to have a good prognosis and might be candidates for studies of reduced ADT. This study suggests that Ki67 is of maximal predictive benefit when used as a continuous variable; this method of stratification is used effectively in the clinic for Ki67 and other expression profiling-based algorithms 19, 22.

Our exclusion of patients with BCR less than 2 years after radiation therapy means the estimates of the predictive value of Ki67 are likely to be conservative. Maximum Ki67 was also a statistically significant predictor of BCR, which is worthy of further study because a single-field assessment of 100 cells is quicker than 4 fields for mean Ki67.

The apparent lack of interaction between Ki67 and fractionation schedule also has clinical implications. The range of proliferative indices seen indicates that the predominantly intermediate-risk PCa included in the trial are usually slowly proliferative. However, when including those cancers showing relatively high proliferation rates, there was no suggestion of a detriment using hypofractionated radiation therapy schedules giving 3 Gy per fraction. Other tumor types encompass wider ranges in proliferation and show higher average proliferation (7). Our results should not be interpreted as demonstrating a general lack of association between proliferation and fraction sensitivity. An important confounding factor may be the complex interplay between fraction sensitivity and overall treatment time (23). Additionally, we acknowledge that the statistical power of tests of interaction are low and that a relatively small proportion of high-risk PCa were included in CHHiP.

Of 3216 patients recruited to CHHiP, 3112 (96.7%) were treated with ADT from just after their diagnostic biopsy until completion of radiation therapy. Androgen-deprivation therapy may modulate fraction sensitivity because it can markedly reduce proliferation and affect repair of double-stranded DNA breaks (24). Androgen-deprivation therapy can also inhibit the cell cycle at the G1/S checkpoint as part of induction of senescence 25, 26. This inhibition could restrict use of double-stranded DNA repair pathway homologous recombination, which operates exclusively in S and G2 and is thought to mediate resistance to fraction sensitivity 27, 28. Cells would instead rely on error-prone nonhomologous end-joining, which operates throughout the cell cycle and is important for fraction sensitivity 27, 28. In the PROFIT trial of radiation therapy fractionation, men did not receive ADT and outcomes were similar to those in CHHiP (29). This suggests that ADT does not have a major impact on average fraction sensitivity in PCa; however, ADT may have confounded the interaction between proliferation and BCR according to fractionation schedule in our study.

Our results are supported by a recent report by Pollack et al (30). In this, a single cut-point (11.3%) was used to score Ki67, fractionation schedules differed from those in our study, and there were fewer failure events. However, Ki67 demonstrated independent prediction of prognosis and did not predict fraction sensitivity. It is relevant that Ki67 immunohistochemistry is routinely available and affordable for most pathology laboratories, and automated scoring algorithms are showing potential clinical applicability (31).

This study assessing Ki67 in patients treated with different radiation therapy fractionation schedules reaches 2 conclusions. First, it does not suggest that there is a detriment to using hypofractionated radiation therapy schedules in PCa showing relatively high proliferation. Second, Ki67 is a highly statistically significant biomarker predicting recurrence, independent of established prognostic factors. Because localized PCa shows diverse clinical outcomes, Ki67 has a potential clinical application to guide treatment stratification.
