# Supplementary material for: Ki67 Is an Independent Predictor of Recurrence in the Largest Randomized Trial of 3 Radiation Fractionation Schedules in Localized Prostate Cancer
Source: Int J Radiat Oncol Biol Phys. 2018 Jun 1;101(2):309–15. doi: 10.1016/j.ijrobp.2018.01.072 (PMC5947826; doi:10.1016/j.ijrobp.2018.01.072)
Supplement: Supplementary Material [file mmc1.docx]

**Supplementary appendix**

*Feasibility study to assess tissue microarray (TMA) versus full-face sections (FFS)*

Methods: A pilot study evaluated if TMA could be used for immunohistochemistry. A single TMA was constructed using the checkerboard technique.[11] A 3-4um section was cut from the TMA at 0.5mm into the block. This section and a FFS of each diagnostic core biopsy within the TMA were stained for Ki67 as below. Ki67 was scored in 3 bins (<5%, 5-10%, >10% positive cells). Ki67 scores from the TMA and corresponding FFS for each patient were assessed for agreement using the kappa statistic.

Results: Overall agreement between Ki67 scores using biopsy TMA and FFS was less than 20%. Where both slides were available, agreement was 42% (kappa=0.14, SE=0.16) (tables 1 and 2 below). FFS were therefore used for the subsequent matched case:control study.

**Table E1: Ki67 scores for comparison of tissue microarray (TMA) versus full-face sections (FFS)**

| **TMA discs** | **Number** |
| --- | --- |
| No tissue | 27 |
| No tumour | 19 |
| Too small | 7 |
| Ki67 <5% | 8 |
| Ki67 5-10% | 10 |
| Ki67 >10% | 8 |
| Total | 79 |
| **Full face sections (FFS)** | **Number** |
| No FFS available | 28 |
| No tumour | 1 |
| Repeat IHC needed | 1 |
| H&E review needed | 4 |
| Ki67 <5% | 15 |
| Ki67 5-10% | 20 |
| Ki67 >10% | 10 |
| Total | 79 |

**Table E2: Agreement between full-face sections (FFS) and tissue microarray (TMA) for Ki67 scores**

| **Agreement between scores** |  |  |
| --- | --- | --- |
| Agreement FFS and TMA score | 8 | 17.8% |
| Disagreement FFS and TMA score | 11 | 24.4% |
| Cannot be compared | 26 | 57.8% |
| Total | 45 |  |

**Methodology for IHC staining of Ki67 and CK5/6**

After heat-mediated antigen retrieval in pH6 citrate buffer (20 minutes), slides were placed on a DAKO link Autostainer and stained using a pre-programmed standard two step method. This consisted of 1 hour incubation with Ki-67 antibody (MIB-1 DAKO(M7240), 1:300 dilution), followed by Dako Flex Envision HRP and DAB chromagen. Lymphoid tissue was used as positive control and prostate tissue without primary antibody as negative control. Slides were counterstained in Gills Haematoxylin prior to dehydration, clearing and coverslipping. An adjacent section from each biopsy block was stained with the basal marker CK5/6 (DAKO (D5/16), dilution 1:75, antigen retrieval DAKO PT module pH9.0).

**Figure E1 A: Scatterplot showing concordance in original mean Ki67 between independent scoring investigators, B: Brand-Altman plot showing difference in original scores between investigator 1 and investigator 2 versus means scores.**

**Figure E2: Distribution of the mean scores (2A) and maximum scores (2B) of Ki67 scores in the cases (BCR) and controls (no local recurrence (LR)) by fractionation schedule.**

**A**

**Mean difference* (95% CI)**

1.51 (0.06-2.95), *p=0.04*

2.55 (0.66-4.43), *p=0.009*

1.12 (-0.52-2.76), *p=0.18*

(%)

**Mean difference** (95% CI)**

1.14 (-0.82-3.10), *p=0.25*

3.04 (0.59-5.48), *p=0.02*

1.89 (-0.33-4.11), *p=0.09*

**B**

(%)

***** Difference in the mean of the mean Ki67 scores of paired cases and controls.

****** Difference in the mean of the maximum Ki67 scores of paired cases and controls.

**Table E3 Results of Power calculations**

Note that this power calculation did not account for the matching in the case-control study as such methods for testing an interaction are not available.

| **Comparison** | **Total sample size** | **alpha** | **Power** | **95 % CI** |
| --- | --- | --- | --- | --- |
| 74 Gy vs 60 Gy | 220 | 0.017 | 75.5 | 69.2 – 81.0 |
| 74 Gy vs 57 Gy | 244 | 0.017 | 74.8 | 68.8 – 80.1 |
| 60 Gy vs 57 Gy | 230 | 0.017 | 70.0 | 63.6 – 75.8 |

**Table E4. Odds ratios for BCR estimated from multivariable conditional logistic regression models without and with interaction terms between the maximum Ki67 scores and fractionation schedules.**

| **Schedules** | **Variable** | **OR** | **95 % CI (OR)** | ***P value (OR)*** | ***P value for interaction**** |
| --- | --- | --- | --- | --- | --- |
| **74 Gy & 60 Gy** | max Ki67 | 1.06 | 1.02 – 1.11 | *0.008* | *0.34* |
| **74 Gy & 57 Gy** | max Ki67 | 1.04 | 1.00 – 1.08 | *0.08* | *0.82* |
| **60 Gy & 57 Gy** | max Ki67 | 1.06 | 1.01 – 1.11 | *0.02* | *0.27* |
| OR’s are adjusted for matching variables and age at randomisation. *P value for the interaction between the maximum Ki67 scores and fractionation schedules. | | | | | |

**Hospitals contributing tissue samples to Trans-CHHiP**

| Addenbrooke’s Hospital (55) |
| --- |
| Aintree University Hospital (1) |
| Alexandra Hospital, Redditch (11) |
| Barnsley Hospital NHS Foundation Trust (9) |
| Basingstoke and North Hampshire Hospital (21) |
| Bedford Hospital NHS Trust (56) |
| Belfast City Hospital (16) |
| Birmingham City Hospital (7) |
| Birmingham Heartlands Hospital (2) |
| Bradford Royal Infirmary (5) |
| Bristol Royal Infirmary (1) |
| Burnley General Hospital (4) |
| Charing Cross Hospital (5) |
| Chesterfield Royal Hospital (26) |
| Countess of Chester Hospital (33) |
| Croydon University Hospital (88) |
| Darlington Memorial Hospital (1) |
| Dewsbury and District Hospital (12) |
| Doncaster Royal Infirmary (31) |
| Ealing Hospital, London (4) |
| East Surrey Hospital (15) |
| Eastbourne District General Hospital (36) |
| Epsom Hospital (54) |
| Falkirk Community Hospital (1) |
| Frimley Park Hospital (9)  Furness General Hospital (1) |
| George Eliot Hospital, Nuneaton (4) |
| Gloucestershire Royal Hospital (1) |
| Good Hope Hospital, Birmingham (3) |
| Hereford County Hospital (18) |
| Hillingdon Hospital (2) |
| Huddersfield Royal Infirmary (8) |
| Ipswich Hospital (137) |
| James Paget University Hospital, Great Yarmouth (1) |
| Kent and Sussex Hospital (1) |
| Kettering Hospital (18) |
| Kingston Hospital (70) |
| Lincoln County Hospital (150) |
| Maidstone Hospital (3) |
| Manchester Royal Infirmary (13) |
| Milton Keynes University Hospital (10) |
| Musgrove Park Hospital, Taunton (10) |
| Newham University Hospital (13) |
| NHS Fife (1) |
| NHS Forth Valley (8) |
| NHS Inverclyde (16) |
| Noble’s Hospital, Isle of Man (11) |
| Norfolk & Norwich University Hospital (57) |
| North Staffs University Hospital (21) |
| Northampton General Hospital (19) |
| Northwick Park Hospital, London (29) |
| Princess Alexandra Hospital, Harlow (11) |
| QE Gateshead (4) |
| Queen Elizabeth Hospital, Birmingham (33) |
| Queen Elizabeth Hospital, King's Lynn (55) |
| Queen Elizabeth II Hospital (1) |
| Queen Hospital, Romford (9) |
| Rochdale Infirmary (1) |
| Royal Albert Edward Infirmary, Wigan (2) |
| Royal Blackburn Hospital (30) |
| Royal Bolton Hospital (14) |
| Royal Bournemouth Hospital (6) |
| Royal Free Hospital (2) |
| Royal Lancaster Infirmary (57) |
| Royal London Hospital (17) |
| Royal Marsden Hospital, London (5) |
| Royal Oldham Hospital (9) |
| Royal Preston Hospital (13) |
| Royal Surrey County Hospital, Guildford (16) |
| Royal Sussex County Hospital, Brighton (43) |
| Royal United Hospital, Bath (8) |
| Royal Victoria Hospital, Newcastle (9) |
| Russell’s Hall Hospital (5) |
| Salisbury District Hospital (2) |
| Shirley Oaks Hospital (BMI) (3) |
| Southern General Hospital (2) |
| Southmead Hospital (15) |
| Southport and Formby District General Hospital (48) |
| St. George's Hospital (22) |
| St. Helier's Hospital (26) |
| St. James University Hospital, Leeds (22) |
| St. Marys Hospital, Paddington (6) |
| St. Richard’s Hospital, Chichester (1) |
| Stepping Hill Hospital (6) |
| Stirling Community Hospital (4) |
| Tameside General Hospital (7) |
| The Chase Farm Hospital (2) |
| Torbay Hospital (5) |
| Tunbridge Wells Hospital (1) |
| University Hospital Ayr (10) |
| University Hospital Coventry (40) |
| University Hospital Crosshouse (Glasgow) (42) |
| Wansbeck General Hospital, Northumberland (1) |
| Warrington Hospital (81) |
| Warwick Hospital (19) |
| West Middlesex University Hospital (2) |
| West Suffolk Hospital (73) |
| Western General Hospital, Edinburgh (2) |
| Western Infirmary, Glasgow (4) |
| Weston General Hospital (2) |
| Whipp’s Cross Hospital (24) |
| Whiston Hospital (66) |
| Whittington Hospital (5) |
| Worcester Royal Hospital (10) |
| Wrexham Maelor Hospital (1) |
| Wythenshawe Hospital (6) |
| Yeovil District Hospital (9) |
